# Treatment of adult ALL patients with third-generation CD19-directed CAR T cells: results of a pivotal trial

**DOI:** 10.1186/s13045-023-01470-0

**Published:** 2023-07-22

**Authors:** Maria-Luisa Schubert, Anita Schmitt, Angela Hückelhoven-Krauss, Brigitte Neuber, Alexander Kunz, Philip Waldhoff, Dominik Vonficht, Schayan Yousefian, Lea Jopp-Saile, Lei Wang, Felix Korell, Anna Keib, Birgit Michels, Dominik Haas, Tim Sauer, Patrick Derigs, Andreas Kulozik, Joachim Kunz, Petra Pavel, Sascha Laier, Patrick Wuchter, Johann Schmier, Gesine Bug, Fabian Lang, Nicola Gökbuget, Jochen Casper, Martin Görner, Jürgen Finke, Andreas Neubauer, Mark Ringhoffer, Denise Wolleschak, Monika Brüggemann, Simon Haas, Anthony D. Ho, Carsten Müller-Tidow, Peter Dreger, Michael Schmitt

**Affiliations:** 1 Department of Internal Medicine V, University Hospital Heidelberg, Im Neuenheimer Feld 410, 69120 Heidelberg, Germany; 2 Heidelberg Institute for Stem Cell Technology and Experimental Medicine (HI-STEM gGmbH), Heidelberg, Germany; 3 Division of Stem Cells and Cancer, Deutsches Krebsforschungszentrum (DKFZ) and DKFZ–ZMBH Alliance, Heidelberg, Germany; 4 Faculty of Biosciences, Heidelberg University, Heidelberg, Germany; 5 Berlin Institute of Health (BIH) at Charité – Universitätsmedizin Berlin, Berlin, Germany; 6 Berlin Institute for Medical Systems Biology, Max Delbrück Center for Molecular Medicine in the Helmholtz Association, Berlin, Germany; 7 Department of Hematology, Oncology and Tumor Immunology, Charité University Medicine, Berlin, Germany; 8 Department of Pediatric Hematology, Oncology and Immunology, University Hospital Heidelberg, Heidelberg, Germany; 9 Institute for Clinical Transfusion Medicine and Cell Therapy (IKTZ), German Red Cross Blood Service Baden-Württemberg-Hessen, Heidelberg, Germany; 10 Institute of Transfusion Medicine and Immunology, Medical Faculty Mannheim, of the Heidelberg University, German Red Cross Blood Service Baden-Württemberg – Hessen, Mannheim, Germany; 11 GRN MVZ Sinsheim, Sinsheim, Germany; 12 Department of Internal Medicine II, University Hospital Frankfurt, Frankfurt, Germany; 13 Department of Hematology and Oncology, University Hospital Oldenburg, Oldenburg, Germany; 14 Department of Hematology and Oncology, Hospital Bielefeld, Bielefeld, Germany; 15 Department of Internal Medicine I, University Hospital Freiburg, Freiburg, Germany; 16 Department of Hematology, Oncology and Immunology, University Hospital Giessen und Marburg, Marburg, Germany; 17 Städtisches Klinikum Karlsruhe, Karlsruhe, Germany; 18 Department of Hematology and Oncology, Center of Internal Medicine, Otto-von-Guericke University Medical Center, Magdeburg, Germany; 19 Department of Internal Medicine II, University Hospital Kiel, Kiel, Germany; 20 German Cancer Consortium (DKTK) and German Cancer Research Center (DKFZ)/National Center for Tumor Diseases (NCT), Heidelberg, Germany

**Keywords:** Acute lymphoblastic leukemia (ALL), Third-generation chimeric antigen receptor (CAR) T cells, Investigator-initiated trial (IIT), CART-associated toxicities, Cytokine release syndrome (CRS), Cytopenia, Immune effector cell-associated neurotoxicity syndrome (ICANS), CD39

## Abstract

**Background:**

Third-generation chimeric antigen receptor (CAR)-engineered T cells (CARTs) might improve clinical outcome of patients with B cell malignancies. This is the first report on a third-generation CART dose-escalating, phase-1/2 investigator-initiated trial treating adult patients with refractory and/or relapsed (r/r) acute lymphoblastic leukemia (ALL).

**Methods:**

Thirteen patients were treated with escalating doses of CD19-directed CARTs between 1 × 10^6^ and 50 × 10^6^ CARTs/m^2^. Leukapheresis, manufacturing and administration of CARTs were performed in-house.

**Results:**

For all patients, CART manufacturing was feasible. None of the patients developed any grade of Immune effector cell-associated neurotoxicity syndrome (ICANS) or a higher-grade (≥ grade III) catokine release syndrome (CRS). CART expansion and long-term CART persistence were evident in the peripheral blood (PB) of evaluable patients. At end of study on day 90 after CARTs, ten patients were evaluable for response: Eight patients (80%) achieved a complete remission (CR), including five patients (50%) with minimal residual disease (MRD)-negative CR. Response and outcome were associated with the administered CART dose. At 1-year follow-up, median overall survival was not reached and progression-free survival (PFS) was 38%. Median PFS was reached on day 120. Lack of CD39-expression on memory-like T cells was more frequent in CART products of responders when compared to CART products of non-responders. After CART administration, higher CD8 + and γδ-T cell frequencies, a physiological pattern of immune cells and lower monocyte counts in the PB were associated with response.

**Conclusion:**

In conclusion, third-generation CARTs were associated with promising clinical efficacy and remarkably low procedure-specific toxicity, thereby opening new therapeutic perspectives for patients with r/r ALL.

*Trial registration* This trial was registered at www.clinicaltrials.gov as NCT03676504.

**Supplementary Information:**

The online version contains supplementary material available at 10.1186/s13045-023-01470-0.

## Introduction

Chimeric antigen receptor T cell (CART) products targeting CD19 have been approved for the treatment of patients with relapsed and/or refractory (r/r) B cell malignancies [1–4], including patients with acute lymphoblastic leukemia (ALL) [2, 3].

All commercially available CARTs express second-generation CARs that contain one costimulatory domain. Third-generation CARTs comprising two costimulatory domains have shown superior engraftment, improved expansion capacity and prolonged persistence [5–9]. Clinically, superior expansion and longer persistence were observed when CD19-directed second (CD28 costimulatory domain)- and third-generation (CD28 and 4-1BB) CARTs were simultaneously administered to lymphoma patients [10]. However, clinical data evaluating solely third-generation CARTs are limited [11].

Here, we describe the first results obtained with an academically developed third-generation CAR in the framework of the investigator-initiated trial (IIT) Heidelberg CAR number 1 (HD-CAR-1) in adult patients with r/r ALL. All steps of treatment including leukapheresis, manufacturing and administration of CARTs, patient monitoring as well as patient follow-up were performed in-house.

A solely academic-driven trial, a third-generation Good Manufacturing Practice (GMP) grade retroviral vector and treatment of adult r/r ALL patients with escalating CART doses make this trial hitherto unique.

## Methods

### Study design

Adult patients with confirmed CD19-positive, minimal residual disease (MRD)-positive, hematological or extramedullary r/r ALL received escalating doses of autologous T-lymphocytes retrovirally transduced with a third-generation CD19-directed CAR (RV-SFG.CD19.CD28.4-1BBzeta) [12]. Endpoints included feasibility of manufacturing and treatment safety, clinical efficacy and survival. Patients were evaluated as outlined in the study calendar [12]. Written informed consent was obtained from all patients prior to treatment. The trial was conducted according to the principles of the Declaration of Helsinki.

### HD-CAR-1 CART manufacturing

As described [12, 13], patients underwent leukapheresis for collection of peripheral blood mononuclear cells (PBMCs). PBMCs were transduced with RV-SFG.CD19.CD28.4-1BBzeta retroviral vector supernatant supernatant (provided by Prof. Malcolm Brenner, Baylor College of Medicine, Houston, Texas, USA) after three days of activation with anti-CD3 and anti-CD28 antibodies (MACS GMP Pure, Miltenyi Biotec, Bergisch Gladbach, Germany). RV-SFG.CD19.CD28.4-1BBzeta carries an anti-CD19 scFv derived from the FMC63 antibody inserted to the SFG retroviral backbone. The transmembrane domain is derived from CD28, the hinge domain from the human IgG1-CH2CH3 domain and 4-1BB is inserted between the CD28 and CD3ζ (CAR structure displayed in Additional file 1: Figure S1). Transduced cells were cultured in complete medium supplied with interleukine (IL)-7 (10 ng/mL) and IL-15 (5 ng/mL) (CellGenix, Freiburg, Germany) for a total of 13 days at the GMP Core Facility of the Internal Medicine V Department of the Heidelberg University Hospital (were evaluated) as descibed [12]. Medium change was performed on days 7 and 10. Cells were cryopreserved using an automated device (Biofreeze BV 40 Consarctic, Westerngrund, Germany). Transduction efficiency was assessed using flow cytometry (FACS Canto, BD Biosciences, Franklin Lakes, NJ, USA). After testing for sterility (Ph. Eur. 2.6.1), mycoplasma (Ph. Eur. 2.6.7) and endotoxin (Ph. Eur. 2.6.14), products were released for administration.

### CART treatment and follow-up, evaluation of toxicity and outcome

Patients received the respective dose of HD-CAR-1 CARTs on day 0 after lymphodepletion (fludarabine 90 mg/m^2^ and cyclophosphamide 1500 mg/m^2^). Cytokine release syndrome (CRS) and immune effector cell-associated neurotoxicity syndrome (ICANS) were graded according to the consensus guidelines of the American Society for Transplantation and Cellular Therapy (ASTCT) [14] and managed according to institutional guidelines and as published [15]. Tumor lysis syndrome (TLS) was graded as described [16]. Adverse events (AEs) were graded according to the National Cancer Institute Common Terminology Criteria for Adverse Events (CTCAE), version 5.0. B cell aplasia was defined as B cell count in the PB below 100/µl as assessed by flow cytometry. Lymphodepletion, CART administration and safety monitoring were performed as inpatient procedures with mandatory hospitalization from day -6 through day + 14. Following patient discharge, patients presented in the outpatient department according to the study visit schedule [12].

Clinical efficacy of HD-CAR-1 treatment was assessed according to response criteria defined for ALL [17, 18], i.e., bone marrow (BM) aspiration and/or radiologic imaging in case of extramedullary disease.

### Assessment of CART frequencies

HD-CAR-1 CART frequencies were quantified by single-copy gene (SCG)-based duplex quantitative PCR (SCG-DP-PCR) amplifying simultaneously the human SCG ribonuclease (RNase) P RNA component H1 (RPPH1) and the FMC63 domain of the CAR transgene as described [19].

### Assessment of cellular composition of CART products and patient samples

#### Flow cytometry

From ten HD-CAR-1 patients (unique patient number (UPN)#1-#7 and UPN#9-#11), PBMCs of the manufactured CART product and of peripheral blood (PB) samples collected after CART treatment were analyzed using 35-parametric spectral flow cytometry analysis. PBMCs derived from buffy coats of three healthy donors served as controls (used antibodies summarized in Additional file 1: Table S1).

#### Computational analysis

Spectral unmixing of obtained data was performed using SpectroFlow (Cytek Biosciences). For general downstream analysis, the R packages Spectre [20], CATALYST [21] and diffcyt [22] were used.

Using the Spectre package, CART product and PBMC data were merged into a single data table, with keywords denoting the sample, group and other metadata added to each row (cell). Since data were acquired over the course of two days, batch alignment was performed by computing quantile conversions using reference samples recorded with each batch, and then applied to the samples in each batch using CytoNorm [23] in Spectre. The batch-corrected values were used for all downstream computations including clustering and differential expression analyses.

Analysis of T cells: For detailed clustering and subset annotation of individual T cell populations (CD4 + and CD8 + T cells), the cluster function from the CATALYST package [21] (version 1.18.1) was used, which performs a FlowSOM clustering and ConsensusClusterPlus metaclustering. Markers that were included for clustering were specified and were dependent on the respective T cell population excluding cells expressing the CAR.

For cellular visualizations, the dimensionality reduction algorithm Uniform manifold approximation and projection (UMAP) [24] was used on downsampled data, taking surface expression of used markers into consideration.

Analysis of CAR T cells: For analysis of CD4 + or CD8 + CARTs, the CD4 + and CD8 + T cell clusters were selected and the surface expression of the CAR detection marker was used to gate CAR + T cells. Within the CD4 + or CD8 + T cell compartments, cells were gated using the same cutoffs for every sample. Due to spectral spillover, different cutoffs for the CD4 + and CD8 + compartment were applied.

For principal component analysis (PCA), the cell-type frequencies for each sample were used as input. Cell-type frequencies were calculated sample-wise by dividing the number of cells of per population by the total number of cells within that sample.

To perform differential expression analysis, the diffcyt package [22] (version 1.14.0) was used. The models and contrast matrices were set up with the createFormula and createContrast functions from the diffcyt package. For the differential abundance analysis, a generalized linear mixed model (GLMM) was used and adjusted *p* values (based on Benjamini–Hochberg [25] method) were returned. For differential expression analysis of CD39 on cells of non-responders or responders, a linear mixed model (LMM) was applied and the unadjusted *p* value was reported.

Differential abundance analyses were performed by calculating the frequency of cells per population out of the total CD45 + cells per sample or the frequency of cells per CD4 + or CD8 + T cell subset out of all CD3 + TCRab + T cells, respectively. For comparisons of responders versus non-responders, the mean frequency for every population in non-responders was calculated. Then, frequencies for every population in responders were divided by the corresponding mean frequency from non-responders as determined in the step before. Likewise, for comparisons of CART recipients versus healthy donors, the mean frequency for every population in healthy donors was calculated. Then, frequencies for every population in CAR recipients were divided by the corresponding mean frequency from non-responders as determined in the step before. Sample-specific fold changes were log_2_-transformed and visualized as boxplots.

### Statistical analysis

Statistics were calculated using Prism Software (GraphPad Software Inc., version 8.2.2). Progression-free survival (PFS) was calculated from the date of CART administration until the date of clinical progression, relapse or death, respectively. Differences between survival curves were descriptively calculated by log-rank testing. A *p* value < 0.05 was considered statistically significant.

## Results

### Patient characteristics

**Fig. 1:**
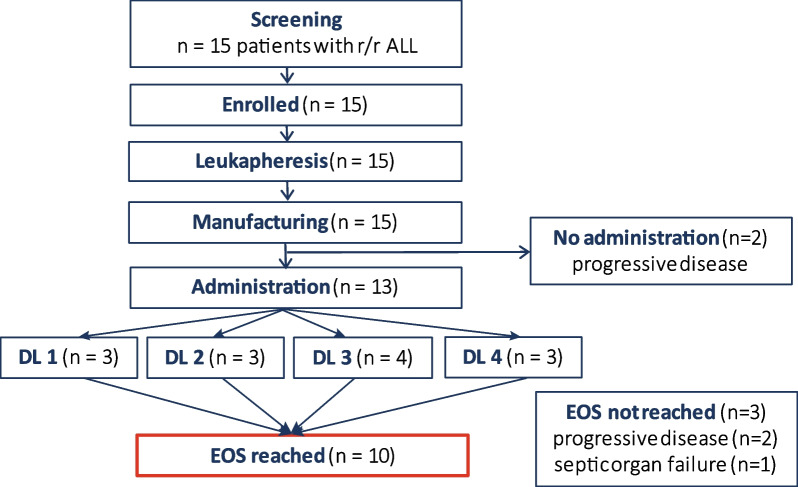
HD-CAR-1 study profile. Fifteen patients with relapsed and/or refractory (r/r) acute lymphoblastic leukemia (ALL) after at least two prior therapy lines were screened and enrolled into HD-CAR-1. For all patients, leukapheresis and manufacturing of CARTs were feasible. Two patients did not receive the HD-CAR-1 CART product due to progressive disease (PD). Thirteen patients were treated with CARTs, with three patients receiving 1 × 10^6^ (dose level (DL) 1), three patients 5 × 10^6^ (DL2), four patients 20 × 10^6^ (DL3) and three patients 5 × 10^7^ (DL4) CARTs/m^2^. Ten patients reached end of study (EOS) on day 90 after CART administration. Three patients died due to progressive disease (*n* = 2) or due to septic organ failure (*n* = 1) prior to EOS

Between September 2018 and January 2022, 15 patients with r/r ALL were enrolled (Fig. 1). The patient baseline characteristics are detailed in Table 1. Median age of patients was 41 (range 21–67) years. Median time from initial diagnosis to CART administration was 22 (range 5–117) months, and patients had received a median of 4 (range 2–9) prior treatment lines, including allogeneic stem cell transplantation (alloSCT) in 12 patients (80%). None of the patients received immune suppression at the time of leukapheresis or had signs of active graft-versus host disease (GvHD). All patients were complete donor chimeras at the time of leukapheresis.

**Table 1:** Patient characteristics

UPN	Age	Gender	Disease	Extra-medullary disease prior CARTs	Diagnosis to CARTs [months]	# Prior tx lines	Prior alloSCT/donor/source	Prior blina	Bridging*	Disease status at LD
1	32	F	B-ALL	Yes (CNS)	117	9	Yes (2×)/2× MUD/2 × PBSCT	Yes	Yes	CSF: 7 blasts /µl; BM: <5% blasts, MRD -
2	21	M	Pre-B-ALL	No	15	4	Yes/MUD/PBSCT	Yes	No	Molecular relapse (BM MRD 0.3%)
3	67	F	B-ALL	No	15	2	No	No	No	Molecular relapse (BM MRD 0.3%)
4	32	F	B-ALL	No	8	2	Yes/MUD/PBSCT	No	No	6% blasts in BM
5	63	M	Pre-B-ALL	No	20	4	Yes/haplo/BMT	Yes	No	8% blasts in BM
6	28	F	B-ALL	Yes (bone)	22	7	Yes/MUD/PBSCT	No	No	PD (right epicondyle, humerus)
7	67	F	MPAL	No	97	4	Yes/MUD/PBSCT	No	Yes	Molecular relapse (BM MRD 0.018%)
8	36	F	Pre-B-ALL	Yes (left mamma, parotid gland, lymph nodes (axillary, mediastinal, parailiacal, inguinal)	8	5	No	Yes	Yes	48% blasts in BM and PD of extramedullary disease
9	45	M	B-ALL	No	25	6	Yes/MUD/PBSCT	No	No	Molecular relapse (BM MRD 0.36%)
10	47	F	B-ALL	No	18	4	Yes/MUD/PBSCT	Yes	No	Molecular relapse (BM MRD 0.3%)
11	37	M	B-ALL	No	52	5	Yes/MUD/PBSCT	Yes	No	BM blasts 6%
12	36	M	B-ALL	Yes (kidney, liver, bone)	28	4	Yes/MMUD/PBSCT	Yes	No	PR of extramedullary manifestations, BM: <5% blasts
13	32	M	B-ALL	Yes (bone)	117	4	Yes/MUD/PBSCT	Yes	Yes	PD of extramedullary manifestations (10^th^ rib, both femurs, both tibias, left Os ilium), BM: <5% blasts
14^§^	47	M	B-ALL	No	41	4	Yes/MMUD/PBSCT	Yes	Yes	Molecular relapse (BM MRD 0.3%)/death due to PD prior LD
15^§^	65	M	B-ALL	No	5	3	No	Yes	Yes	8% blasts in BM/death due to sepsis prior LD

*ALL* acute lymphoblastic leukemia, *alloSCT* allogeneic stem cell transplantation, *B-ALL* common B cell leukemia, *BM* bone marrow, *BMT* bone marrow transplant, *CART* chimeric antigen receptor T cells, *CNS* central nervous system, *CR* complete remission, *CSF* cerebrospinal fluid, *F* female, *haplo* haploidentical hematopoietic stem cell transplantation, *LD* lymphodepletion, *M* male, *MPAL* mixed-phenotype acute leukemia, *MRD* minimal residual disease, *MMUD* HLA-mismatched unrelated donor, *MUD* HLA-matched unrelated donor, *PBSCT* peripheral blood stem cell transplant, *PR* partial remission, *pre-B-ALL* precursor B cell acute lymphoblastic leukemia, *PD* progressive disease, *SD* stable disease, *tx* therapy, *UPN* unique patient number

*Treatment administered between leukapheresis and lymphodepletion

^§^Patients #14 and #15 did not receive HD-CAR-1 CARTs

### Feasibility of HD-CAR-1 CART manufacturing

Leukapheresis and manufacturing of CARTs were successful for all enrolled patients. Due to low T cell counts in the PB of one patient (UPN#12), two consecutive CART production cycles had to be performed. Median duration of CART manufacturing was 10 (range 10–14) days. Median transduction efficiency was 52.7% (range 39.3–66.9%) with a viability of CARTs of > 85%. CART production details are summarized in Additional file 1: Table S2.

### CART administration

Of 15 patients, six patients received bridging therapy between leukapheresis and lymphodepleting therapy. Thirteen patients received HD-CAR-1 CARTs. (UPN#14 and UPN#15 did not receive CARTs due to progressive disease (PD) during CART manufacturing.) Three patients were treated with CARTs at dose level (DL) 1 (1 × 10^6^ CARTs/m^2^), DL2 (5 × 10^6^ CARTs/m^2^) and DL4 (5 × 10^7^ CARTs/m^2^). Four patients were treated at DL3 (2 × 10^7^ CARTs/m^2^) (Fig. 1). Ten patients reached end of study (EOS) on day 90 after CARTs. Three patients did not reach EOS due to PD (*n* = 2) at day 23 (UPN#8) and day 76 (UPN#3), respectively, and due to fatal septic organ failure (*n* = 1; UPN#12) on day 39 (Table 2).

**Table 2:** Toxicity and clinical response to treatment with the HD-CAR-1 product

UPN	Age	Gender	Disease	CART dose [CARTs/m^2^]	CRS grade	ICANS	Infection	Best response	Response at EOS	alloSCT after CARTs	Progression of disease/death [day after CARTs]
1	32	F	B-ALL	1 × 10^6^	I	–	RSV	PD	PD	–	28 (CSF: 13 blasts /µl)/254
2	21	M	Pre-B-ALL	1 × 10^6^	–	–	–	CR MRD +	CR MRD +	Yes	119 (BM: 8% blasts)/268
3^†^	67	F	B-ALL	1 × 10^6^	–	–	–	CR MRD + (day 28)	n.r	–	56 (BM: 52% blasts)/76
4	32	F	B-ALL	5 × 10^6^	–	–	–	CR MRD −	CR MRD −	–	–
5	63	M	Pre-B-ALL	5 × 10^6^	–	–	*Staph. epi*	CR MRD −	CR MRD +	–	146 (extramedullary: malignant ascites)
6	28	F	B-ALL	5 × 10^6^	I	–	–	CR MRD −	CR MRD-(metabolic CR)	–	120 (extramedullary: right mamma)
7	67	F	MPAL	20 × 10^6^	–	–	–	CR MRD −	CR MRD +	–	–
8^†^	36	F	Pre-B-ALL	20 × 10^6^	II	–	–	PD	n.r	Yes	23 (BM: 100% blast infiltration)/58
9	45	M	B-ALL	20 × 10^6^	–	–	–	CR MRD −	CR MRD −	–	189 (CSF: 50–60% blasts)
10	47	F	B-ALL	20 × 10^6^	–	–	–	CR MRD +	PD	Yes	93 (BM: 9% blasts)
11	37	M	B-ALL	50 × 10^6^	–	–	Respiratory*	CR MRD −	CR MRD −		
12^†^	36	M	B-ALL	50 × 10^6^	II	–	*E. coli* sepsis	SD (day 26)	n.r	–	–/39 (TRM: septic MOV)
13	32	M	B-ALL	50 × 10^6^	–	–	Respiratory*	CR MRD −	CR MRD-(metabolic CR)	–	–

*ALL* acute lymphoblastic leukemia, *BM* bone marrow, *c-ALL* common B cell leukemia, *CART* chimeric antigen receptor T cells, *CR* complete remission, *CRS* cytokine release syndrome, *CSF* cerebrospinal fluid, *EOS* end of study on day 90 after HD-CAR-1 treatment, *F* female, *M* male, *MPAL* mixed-phenotype acute leukemia, *MOV* multi-organ failure, *MRD* minimal residual disease, *n.r*. not reached, *pre-ALL* precursor B cell acute lymphoblastic leukemia, *PD* progressive disease, *RSV* respiratory syncytial virus, *SD* stable disease, *TRM* treatment-related mortality, *UPN* unique patient number

^†^Patient died before reaching EOS

*No pathogen identified

### Safety

#### CRS/ICANS

Treatment with HD-CAR-1 CARTs was well tolerated (Table 2): None of the patients developed ICANS. Two patients (UPN#1 and UPN#6) developed grade I CRS with symptoms limited to fever that resolved with supportive treatment only and two patients (UPN#8, UPN#12) developed grade II CRS and received treatment with tocilizumab. UPN #8 additionally received steroids. One patient (UPN#5) developed febrile temperatures eventually attributable to *Staph. epidermidis* bacteremia that resolved with targeted antibiotic treatment (Additional file 1: Table S3). Higher-grade CRS, i.e., grade ≥ III, was not observed in any patient.

Patient #8, who suffered from multiple extramedullary ALL lesions prior treatment (Table 1), developed fever and mild hypoxia three days after CART administration suggestive for CRS. With high ferritin, triglycerides and sCD25, a concomitant hemophagocytic lymphohistiocytosis (HLH)/macrophage activation syndrome (MAS) was suspected. Several doses of tocilizumab and steroids were administered. Due to further clinical deterioration including kidney failure, eventually PD was suspected. A BM biopsy performed on day 10 confirmed PD with almost 100% infiltration of the BM with ALL blasts. CRS symptoms and kidney failure were retrospectively attributed to an overlapping TLS. The patient was subsequently treated with inotuzumab-ozogamicin and received an alloSCT from a matched-related donor, but eventually died 58 days after CART infusion.

Patient #12 received CARTs 16 months after a mismatched unrelated donor alloSCT that had been complicated by a grade 3 gastrointestinal (GI) GvHD [26]. On day 8, the patient developed CRS grade II and received tocilizumab. On day 19, the patient displayed an enterocolitis that evolved into an *E. coli* sepsis and progressed to septic shock with disseminated intravascular coagulation (DIC) and multi-organ failure. The patient died on day 39. Autopsy revealed that the enterocolitis had most probably been provoked and aggravated by GI-GvHD exacerbation. Elevated CART expansion was neither observed in the afflicted parts of the gut mucosa, nor in ascites/pleural effusion as proven by post-mortem quantitative PCR.

#### Cytopenia, B cell aplasia and infectious complications

**Fig. 2:**
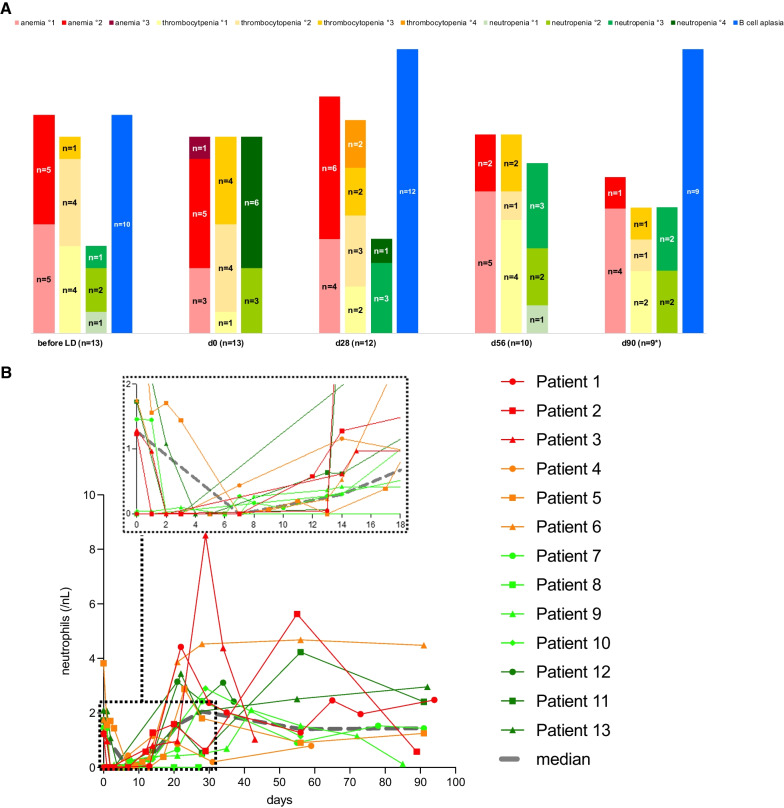
Hematologic toxicity of HD-CAR-1 treatment. **A** Cytopenia and B cell aplasia. On day 0, i.e., after lymphodepletion (LD) and before CART administration, 69% (*n* = 9) of patients were neutropenic (46% grade IV neutropenia), anemic (8% grade III anemia) and thrombocytopenic (31% grade III thrombocytopenia). One month after HD-CAR-1 treatment, 8% (*n* = 1, UPN#4) patients displayed grade IV neutropenia and 17% (*n* = 2; UPN#4 and UPN#11) grade IV thrombocytopenia. On end-of-study (EOS) at day 90, two patients showed persistent grade III neutropenia (UPN#2, UPN #4) and one patient grade III thrombocytopenia (UPN#4) despite treatment with granulocyte-colony-stimulating factor (G-CSF) and a thrombopoietin-agonist, respectively. (UPN#4 had grade III neutropenia and thrombocytopenia already before receiving CARTs; also, UPN#2 was already neutropenic before CART treatment.) No higher-grade anemia was observed. Beyond day 28, no grade IV cytopenia was observed. As for B cell counts, 77% (*n* = 10) of patients displayed B cell aplasia already before receiving CARTs. At EOS, all evaluable patients (*n* = 9; UPN#9 not shown due to PD) had ongoing B cell aplasia (B cell count on day 0 and day 56 not assessed). **B** Absolute neutrophil count (ANC) of treated HD-CAR-1 patients (*n* = 13) within the first 18 days (top, small frame) and up to end of study on day 90 after CART treatment. Four patients (UPN #1, #3, #4, #11, #13) received G-CSF after CARTs. Median of ANCs is depicted in grey

Cytopenia, B cell aplasia and recovery of neutrophil counts are depicted in Fig. 2A, B. Persistent, i.e., beyond EOS, high-grade (≥ III) neutropenia was observed in two of nine evaluable patients (22%). Grade III neutropenia and thrombocytopenia were observed in UPN#4 who had low blood counts already before CARTs. Grade III neutropenia in UPN#2 persisted until an alloSCT, which was performed on day 126. No higher-grade anemia was observed.

Ten patients (77%) had low B cell counts already prior CARTs, most likely due to pretreatment with blinatumomab (*n* = 8) or cytoreductive bridging treatment (*n* = 4) (Table 1). At EOS, all evaluable patients (*n* = 9; UPN#9 not shown due to PD at EOS) had ongoing B cell aplasia (Fig. 2A), even though recovered levels of immunoglobulins were detectable in six patients (data not shown).

Until EOS, two patients (UPN#11 and UPN#13) developed respiratory infections that required oral antibiotics. One patient (UPN#1) was diagnosed with respiratory syncytial virus (RSV) that resolved with supportive care only. No patient with a prior alloSCT reactivated with cytomegalovirus (CMV) or Epstein–Barr virus (EBV).

#### Other toxicities

Two patients who had undergone a prior alloSCT developed GvHD after CART treatment. UPN#6 displayed a GvHD of the lung, and a preexisting GvHD of the GI tract exacerbated in UPN#12 (see above). No non-hematologic (≥ grade III toxicity not preexisting or attributed to underlying malignancy) or hematologic (grade IV cytopenia (except lymphopenia) persisting beyond day 30 post-CARTs) dose-limiting toxicity (DLT) occurred. Toxicities after CAR T cells showed no association with the administered CAR T cell dose. Toxicities are summarized in Additional file 1: Table S3.

### Outcomes

**Fig. 3:**
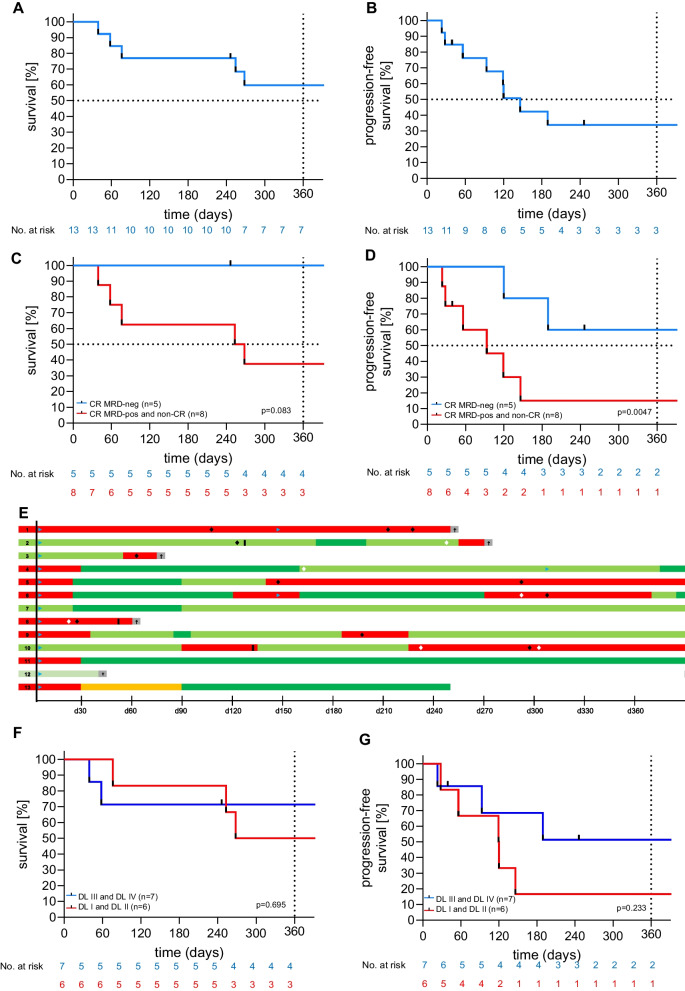
Efficacy of HD-CAR-1 treatment and patient outcome. **A** Overall survival (OS) and **B** progression-free survival (PFS) of treated patients. **C** OS and **D** PFS at end of study (EOS) on day 90 after HD-CAR-1 CART administration of HD-CAR-1 patients that achieved complete remission (CR; blue) vs. non-responders (red; partial remission (PR), stable disease (SD), progressive disease (PD). **E** Swimmer plot depicting the course of individual HD-CAR-1 patients. **F** OS and **G** PFS according to administered HD-CAR-1 CART dose (dose level (DL); DL1: 1 × 10^6^ CARTs/m^2^ (*n* = 3), DL2 5 × 10^6^ CARTs/m^2^ (*n* = 3), DL3 20 × 10^6^ CARTs/m^2^ (*n* = 4), DL4: 5 × 10^7^ CARTs/m^2^ (*n* = 3)). DL: dose level; CR: complete remission; and MRD (minimal residual disease).  : CART therapy.  : allogeneic stem cell transplantation.  : antibody treatment.  : chemotherapy.  : progressive disease (PD),  : partial remission (PR),  : stable disease (SD),  : MRD-positive complete remission (CR),  : MRD-negative complete remission/metabolic CR (CR*), †: death

Overall, ten patients (77%) achieved a complete remission (CR) as best response. Seven patients (54%) attained MRD negativity. At EOS, 10 patients were evaluable for response: eight patients (80%) achieved CR, with five patients (50%) confirmed to be MRD-negative. Median overall survival (OS) at 12-month follow-up was not reached (Fig. 3A), and median PFS was reached on day 120 (Fig. 3B). In patients who achieved a MRD-negative CR at EOS (*n* = 5), 100% OS (Fig. 3C) and 60% PFS (Fig. 3D) at 1-year follow-up were higher compared to patients that failed MRD clearance (*n* = 8) (OS 38%; PFS 12.5%; OS *p* = 0.08; PFS *p* = 0.0047). Three patients received a second CART administration (UPN#1, #4, #6) that eventually mediated MRD-negative CR in two patients (UPN#4 and UPN#6). Three patients underwent alloSCT after HD-CAR-1 treatment (first alloSCT, *n* = 1 (UPN#8); second alloSCT, *n* = 2 (UPN#2, UPN#10)). Evolution of patients within one year after HD-CAR-1 treatment is depicted in Fig. 3E. Of the patients that reached EOS (*n* = 10), two patients have died from PD (UPN#1) and complications after an alloSCT (UPN#2), respectively, two patients are alive with disease (UPN #5,10), two patients are in MRD-positive (UPN#7,9) and four patients in MRD-negative CR (UPN#4,6,11,13). With exception of UPN#6, relapses remained positive for CD19 expression (UPN#3 not assessed for CD19 status at relapse).

Response to treatment was associated with CART doses: Patients that were treated with higher CART doses, i.e., DL3 and DL4, showed a trend toward superior OS (Fig. 3F) and PFS (Fig. 3G) compared to patients that received lower CART doses, i.e., DL1 and DL2.

### CART expansion

**Fig. 4:**
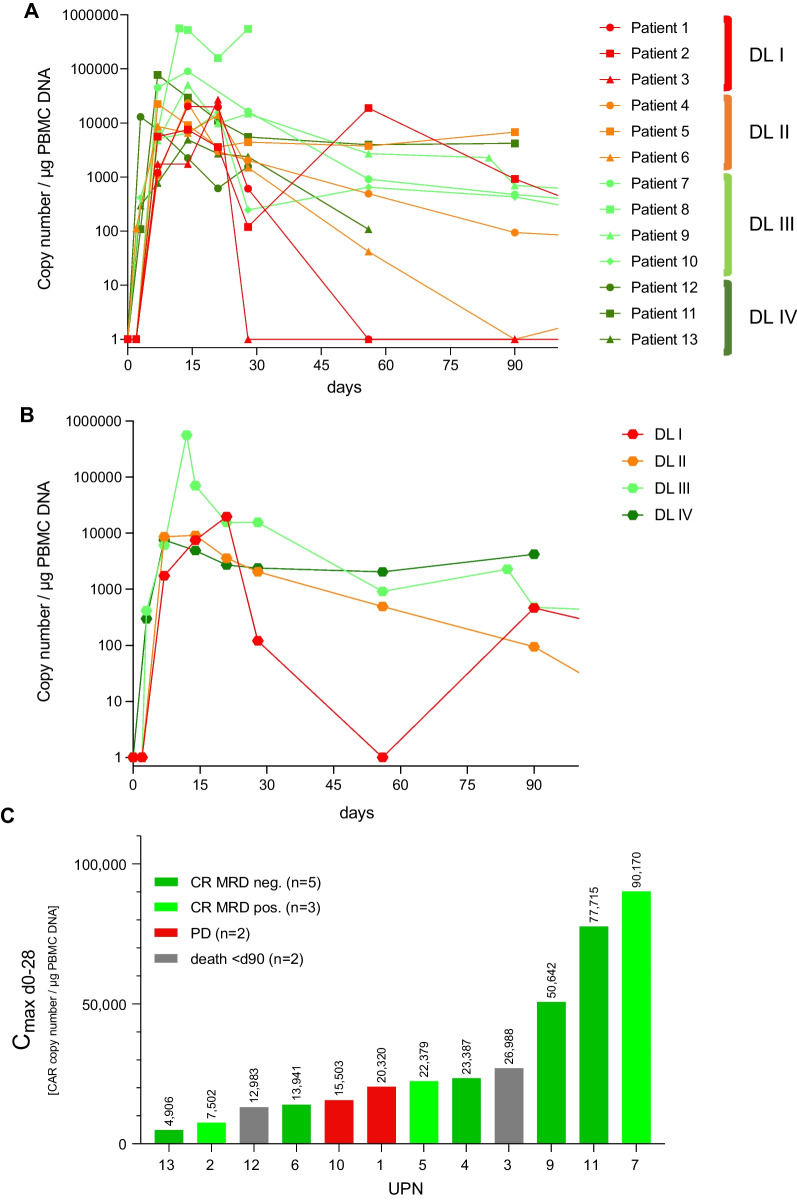
Expansion of HD-CAR-1 CARTs. **A** Expansion of CARTs in the peripheral blood (PB) of individual HD-CAR-1 patients (*n* = 13) assessed by single-copy gene duplex quantitative PCR (SCG-DP-PCR) [19] after CART administration and up to end of study (EOS) at day 90. **B** Median expansion of CARTs according to administered CART dose levels (DL; DL1: 1 × 10^6^ CARTs/m^2^, DL2: 5 × 10^6^ CARTs/m^2^, DL3: 20 × 10^6^ CARTs/m^2^, DL4: 5 × 10^7^ CARTs/m^2^). **C** Maximum CART copies (c_max_) within 28 days after CART administration and clinical response at EOS (data of UPN#8 not shown due to progressive disease on day 23 after CARTs). Median (c_max_) 22.350 CART/µg DNA PBMC. *CR* complete remission, *d* day, *DL* dose level, *MRD* minimal residual disease, *PBMC* peripheral blood mononuclear cell, *PD* progressive disease, *UPN* unique patient number

PB CART expansion was observed in all patients immediately after CART administration. At EOS, CARTs were still detectable in seven (78%) of the evaluable patients (Fig. 4A). Higher CART doses (DL3, DL4) resulted in higher and prolonged expansion levels, whereas loss of detection occurred in patients that had received lower CART doses (DL1: UPN#1, #3, DL2: UPN#6) (Fig. 4B). Patients reaching expansion levels exceeding the median of 22.350 CART/µg DNA PBMC within the first month after treatment were more likely to respond than patients who displayed CART expansion below the median (Fig. 4C).

### Cellular landscape of the CART product and the PB of patients after CART treatment

**Fig. 5:**
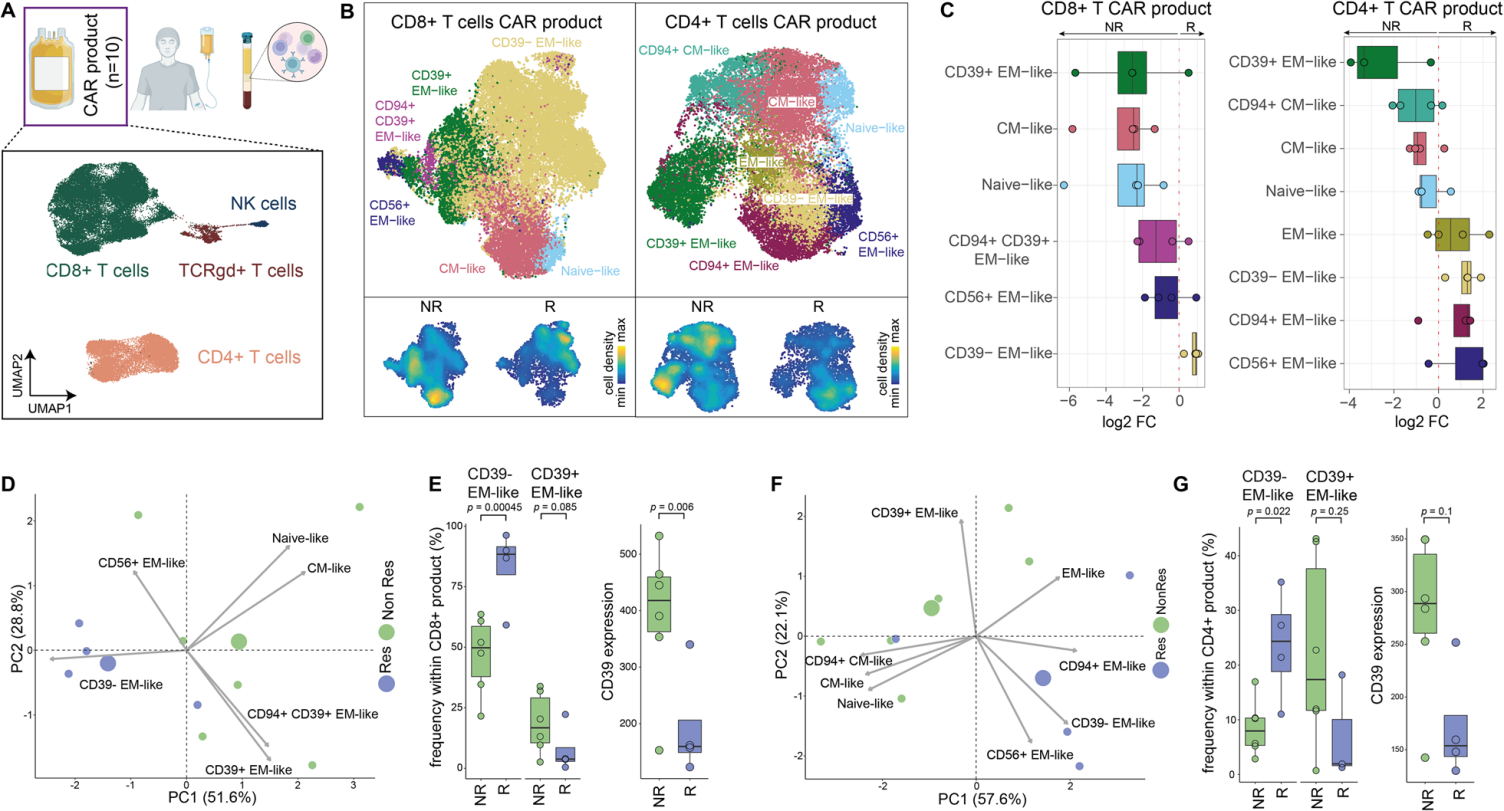
Characterization of the cellular composition of CART products of HD-CAR-1 patients (*n* = 10). **A** CART infusion products were analyzed via high-parametric spectral flow cytometry, and data were analyzed (see methods). Uniform manifold approximation and projection (UMAP) visualization display a downsampled subset of cells from all ten CART products (bottom). After clustering, individual clusters were annotated based on surface marker expression [66] and highlighted by different colors. **B** CD8 + and CD4 + T cell subsets from the CART product of ten patient samples were extracted and clustered separately. A representative subset of cells from all ten CART products is displayed in the UMAP visualizations. Density plots in the two lower panels indicate the differential distribution of cells between non-responders (NR) and responders (R) within the CD8 + and CD4 + T cell compartment, respectively. **C** Boxplots indicating differential abundance of individual clusters from CD8 + (left) and CD4 + T cell (right) subsets from the CART product of responders and non-responders. Positive log_2_ fold changes indicate higher levels in responders, whereas negative log_2_ fold changes indicate that a specific population is more abundant in non-responders. **D** Principal component analysis (PCA) of CD8 + T cells within the CART product. Cell-type frequencies of cell clusters from each sample were used as input for the PCA. Blue circles represent samples from responders, and green circles represent samples from non-responders. The two larger circles indicate the midpoint of the respective group. Gray arrows indicate the variables. **E** Boxplots indicating the abundance of CD39- effector memory (EM)-like and CD39 + EM-like cells within the CD8 + T cell population of the CART products (left). A generalized linear mixed model (GLMM) was used to compute significance between non-responders and responders. Adjusted *p* values are shown. Boxplot of CD39 expression levels in non-responders and responders within the CD8 + T cell subset of the CART product is displayed (right). Significance was assessed by applying a linear mixed model (LMM). **F** PCA of CD4 + T cells within the CART product. Cell-type frequencies of cell clusters from each sample were used as input for the PCA. Blue circles represent samples from responders, and green circles represent samples from non-responders. The two larger circles indicate the midpoint of the respective group. Gray arrows indicate the variables. **G** Boxplots showing the abundance of CD39- EM-like and CD39 + EM-like cells within the CD4 + T cell population of the CART product (left). A GLMM was used to compute significance between non-responder and responder. Adjusted *p* values are shown. Boxplot of CD39 expression levels in non-responder and responder samples within the CD4 + T cell subset of the CAR product (right). Significance was assessed by applying a LMM. *R* responders, *NR* non-responders, *CM* central memory T cells, *cDC* conventional dendritic cells, *EM* effector memory T cells, *NK* natural killer

**Fig. 6:**
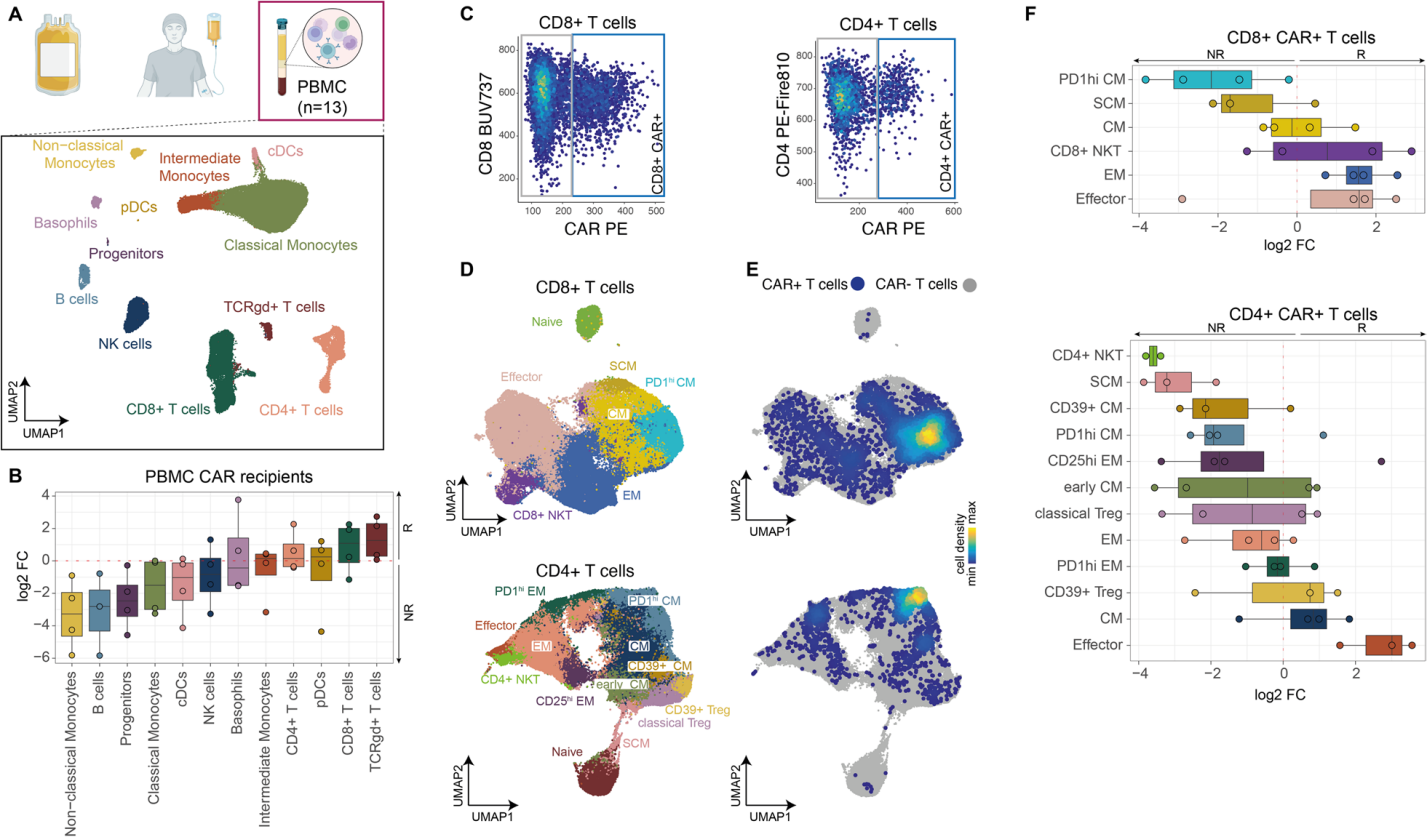
Characterization of the cellular composition of PB samples (*n* = 10) of patients after HD-CAR-1 treatment (*n* = 10) and PB composition of healthy donors (*n* = 3). **A** PBMC samples obtained from patients after CART administration were analyzed via high-parametric spectral flow cytometry and data were analyzed (see methods). UMAP visualization (bottom) showing a downsampled subset of PBMCs from ten CART recipients and additionally three healthy donor samples. After clustering, individual clusters were annotated based on surface marker expression and highlighted by different colors. **B** Boxplots indicating differential abundance of individual cell populations from PBMC samples collected after CART administration, comparing abundances in responders and non-responders. Positive log_2_ fold changes indicate that a respective population is more abundant in responders (R), whereas negative log_2_ fold changes indicate that the population is more abundant in non-responders (NR). **C** Scatterplot displays the gating strategy to define CAR + cells. CD8 + and CD4 + T cells from the PBMC samples were extracted, and fluorescence intensity levels of CD8/CD4 expression were plotted against the fluorescence intensity of the CAR targeting antibody. CAR + cells were determined by setting a CD8 + /CD4 + T cell-specific cutoff for downstream analysis and visualization. **D** UMAP visualizations of downsampled subsets from separately clustered CD8 + and CD4 + T cells identified in **A**. Dimensionality reduction and clustering were performed excluding the expression information of the CAR targeting antibody, to prevent CAR + specific clusters. After clustering, individual clusters were annotated based on surface marker expression and highlighted by different colors. **E** Density plots illustrating the distribution of CAR + cells within the CD8 + T cell (top) and CD4 + T cell (bottom) UMAP embedding. CAR + cells were identified and gated as displayed in **C** and as described in the material and methods section. **F** CD4 + and CD8 + T cells from **D** were used and binned into CAR- and CAR + CD8 + or CD4 + T cells, respectively, as described above (Fig. 5C). Boxplots display differential abundance of different CAR + CD8 + T cells (top) or CAR + CD4 + T cell phenotypes (bottom) of responders and non-responders. Positive log_2_ fold changes indicate that a respective population is more abundant in samples of responders, whereas negative log_2_ fold changes indicate that the population is more abundant in samples of non-responders. *R* responders, *NR* non-responders**,** *CM* central memory T cells, *cDC* conventional dendritic cells, *EM* effector memory T cells, *hi* high, *TCR* T cell receptor, *NK* natural killer, *NKT* natural killer T cells, *pDC* plasmacytoid dendritic cells, *SCM* memory stem cell-like T cells

We used high-dimensional flow cytometry to characterize CART products (Fig. 5) and PB composition (Fig. 6) of treated patients. The CART product of analyzed patients (*n* = 10) contained mostly CD4 + and CD8 + T cells. Also, minor fractions of γδ-T cells and natural killer (NK) cells were identified (Fig. 5A, Additional file 1: Fig. S1A). All CART products contained CAR-positive T cells (Additional file 1: Table S2). Unsupervised clustering and dimensionality reduction of the CD4 + and CD8 + T cell compartments revealed differences in the cellular composition of the CART product in responders and non-responders (Fig. 5B, C): In responders, higher frequencies of CD39-negative effector memory-like CD4 + and CD8 + T cells were observed, whereas non-responders displayed higher levels of CD39-positive effector memory-like T cells (Fig. 5C, D, F). In fact, CD39 expression of all T cells in both CD4 + and CD8 + CART product subsets was elevated in non-responders (Fig. 5E, G), suggesting association of CD39 expression in the CART product with positive therapeutic outcome.

As for analysis of PBMCs obtained after CART administration, non-responders displayed elevated levels of monocytes, whereas responders showed a tendency toward higher CD8 + T cell and γδ-T cell frequencies (Fig. 6A, B). Expectedly, B cells in the highly-pretreated CART recipients were almost completely absent when compared to healthy donors (Additional file 1: S2B and Fig. 2A). Notably, the cellular landscape of patient #11 who remains in ongoing CR after CARTs (without further treatment (Fig. 3E)) was similar to the physiological cellular composition of healthy donors (Additional file 1: S2F). Unsupervised clustering and dimensionality reduction of CD4 + and CD8 + T cell subsets revealed well-known T cell differentiation states of CAR-negative, endogenous T cells and CAR-positive T cells (Fig. 6C, D). Both CD4 + and CD8 + CARTs of responders adopted to a higher degree effector memory and effector T cell states, whereas CARTs of non-responders predominantly adopted central memory phenotypes with high PD-1 expression (Fig. 6E, F). Similar findings were observed in the endogenous T cell compartments (Additional file 1: Fig. S2C–E).

## Discussion

Despite novel therapeutic options for treatment of r/r ALL patients [27–29], the outcome for older patients remains poor [30–32]. Here, we treated adult ALL patients with escalating doses of CD19-specific third-generation CARTs. For all patients, CART products were successfully manufactured. Despite low CART doses administered and a heavily pre-treated patient cohort, a CR rate of 80% including 50% MRD-negative CR was achieved. At 12 months, 38% of evaluable patients remained progression-free and median OS was not reached. Patients who achieved an initial MRD-negative CR did not reach median PFS and were all alive at 1-year follow-up.

The CAR construct used within HD-CAR-1 has already been evaluated in the context of two clinical trials that focused on patients with non-Hodgkin’s lymphoma (NHL). Also, five adult ALL patients (*n* = 1 [10], *n* = 4 [11]) were included. No response in the single ALL patient [10] and CR in 50% (two of four ALL patients) [11] were reported, although results were limited by the small patient number.

Result of HD-CAR-1 is in line with previous trials of second-generation CARTs in adult ALL patients: Park et al. reported a CR rate of 83% (*n* = 53), with 50% OS and 30% event-free survival (EFS) one year after treatment [33]. In the ZUMA-3 trial resulting in the approval of Brexucabtagene autoleucel for treatment of adult r/r ALL patients in 2021, a CR rate of 71% (*n* = 55) with 12-month OS of 75% and EFS of 50% was observed. These results might be at least in part be attributable to the strict exclusion criteria in the ZUMA-3 trial of included patients [34]. Frey et al. reported a CR rate of 69% (*n* = 35) with OS of 47% and EFS of 31% at two-year follow-up. In this latter trial, high-dose (5 × 10^8^), fractionated CART administration (*n* = 20) resulted in unmet median OS and EFS one year after treatment. Of note, only 25% of patients had received a prior alloSCT [35].

Relapses post-CARTs have been reported in 30 to 60% of ALL patients [33, 36–39]. Also in HD-CAR-1, 50% of patients with CR at EOS, have relapsed in the first year after treatment and three patients have received a consecutive alloSCT after HD-CAR-1 treatment. In line with data on the ZUMA-3 trial [34], efficacy of HD-CAR-1 CARTs was limited in patients with extramedullary disease: Only one patient (UPN #13) with extramedullary disease at CART treatment did not relapse after CART treatment, underlining the difficulties of treatment of this patient subgroup.

Of note, HD-CAR-1 appears to be associated with a highly favorable toxicity profile, even at high-dose levels: No ICANS or higher-grade CRS occurred, and only low-grade (I-II°) CRS was observed in 31% of the patients. Prior alloSCT was associated with fatal exacerbation of a most likely preexisting GvHD in one patient. Although an immunogenic effect of CARTs cannot be excluded, GvHD might have been rather triggered substantially by preceding therapies including inotuzumab ozogamicin and blinatumomab.

As for hematotoxicity, the rate of prolonged neutropenia was comparable or lower than the one of previous reports [37, 40, 41], despite the high rate of patients after an alloSCT.

CD28-costimulation has been associated with rapid expansion and marked anti-tumor efficacy [42–44], and 4-1BB has been shown to enhance proliferation, to reduce exhaustion and to mediate long-term CART persistence [45–47]. In fact, we observed fast expansion of HD-CAR-1-CARTs. In contrast to loss of CARTs in the PB of patients treated in ZUMA-3 on day 28 [48], CARTs were durably detected in HD-CAR-1 patients. In line with others, initial MRD-negative response [38, 49] and higher doses of administered CARTs [35] resulted in higher CART frequencies and improved outcome.

High-resolution immunophenotyping revealed an immune cell repertoire of responders characterized by general activation of T cells. In contrast to others, we observed no influence on response by myeloid subtypes [11] or CD4 + /CD8 + T cell ratio [50, 51]. Interestingly, the patient with the most durable response to treatment (UPN#11) displayed a distribution of immune cells in his PB which resembled the cellular composition of the PB of healthy donors. In patients responding to CART therapy, we observed a higher number of γδ T cells within collected PBMCs. In fact, infiltration of malignancies with γδ T cells is associated with favorable prognosis [52] and in the allo-SCT setting, γδ T cells have been associated with enhanced anti-tumor response, improved OS and reduced occurrence of GvHD [53, 54].

Within the CART product, expression of CD39 on effector T cells predicted response: Low levels of this T cell subset were observed in responders, high levels in non-responders. CD39 is expressed on T cell subsets [55, 56], and its expression on CD8-positive T cells has been associated with T cell exhaustion [57, 58]. While CARTs with a less differentiated phenotype, e.g., central memory or naïve CARTs, mediate better expansion, persistence and antitumor activity [59, 60], T cell exhaustion is associated with inferior response [61, 62]. In the context of CARTs, CD39 expression has been linked to reduced CART expansion [63, 64]. Here, we confirm clinically that CD39 within the CART product might be highly relevant to predict outcome in CART patients. In contrast to other molecules such as PD-1 that have been identified not only in CART samples but also in healthy individuals [65], CD39 might constitute a more specific marker for T cell exhaustion.

## Conclusion

In summary, administration of third-generation HD-CAR-1 CARTs was remarkably safe and of promising efficacy. Responses correlated with MRD clearance and were dose-dependent. Lack of CD39 expression on T cell subsets within the CART product was associated with improved anti-leukemic activity of CARTs. HD-CAR-1 appears to be a promising step toward safe and effective ALL eradication in older patients.

## Supplementary Information

**Additional file 1. Table S.1** Antibodies used for assessment of cellular composition of CART products and of PB samples of patients after CART treatment. **Table S.2** Characterization of the leukapheresis and the HD-CAR-1 product by conventional flow cytometry. **Table S.3** HD-CAR-1 adverse events according to CTCAE. **Figure S.1** Structure of the RV-SFG.CD19.CD28.4-1BBzeta retroviral vector. **Figure S.2** Additional data on the cellular composition of CART products and of corresponding PB samples.

## Data Availability

The datasets used and/or analyzed within this study as well as materials are available from the corresponding author on request.

## Abbreviations

AE: Adverse event
ALL: Acute lymphoblastic leukemia
alloSCT: Allogeneic stem cell transplantation
ASTCT: American Society for Transplantation and Cellular Therapy
BM: Bone marrow
CAR: Chimeric antigen receptor
CART: Chimeric antigen receptor T cells
CD: Cluster of differentiation
*c*_max_: Maximal concentration
CMV: Cytomegalovirus
CR: Complete remission
CRS: Cytokine release syndrome
CTCAE: Common Terminology Criteria for Adverse Events
DIC: Disseminated intravascular coagulation
DL: Dose level
DLT: Dose-limiting toxicity
EBV: Epstein–Barr virus
EFS: Event-free survival
EOS: End of study
GI: Gastrointestinal
GLMM: Generalized linear mixed model
GMP: Good manufacturing practice
GvHD: Graft-versus-host disease
HD-CAR-1: Heidelberg CAR trial 1
HLH: Hemophagocytic lymphohistiocytosis
ICANS: Immune effector cell-associated neurotoxicity syndrome
IIT: Investigator-initiated trial
IL: Interleukin
LMM: Linear mixed model
MAS: Macrophage activation syndrome
MRD: Minimal residual disease
NHL: Non-Hodgkin lymphoma
n.r.: Not reached
NR: Non responder
OS: Overall survival
PB: Peripheral blood
PBMC: Peripheral blood mononuclear cell
PCA: Principal component analysis
PD: Progressive disease
PFS: Progression-free survival
PR: Partial remission
R: Responder
RSV: Respiratory syncytial virus
r/r: Refractory and/or relapsed
SD: Stable disease
SCG-DP-PCR: Single-copy gene-based duplex quantitative PCR
TLS: Tumor lysis syndrome
UKHD: Heidelberg University Hospital
UMAP: Uniform manifold approximation and projection
UPN: Unique patient number
