# Supplementary material for: Treatment of adult ALL patients with third-generation CD19-directed CAR T cells: results of a pivotal trial
Source: J Hematol Oncol. 2023 Jul 22;16:79. doi: 10.1186/s13045-023-01470-0 (PMC10363324; doi:10.1186/s13045-023-01470-0)
Supplement: Supplementary file 1 — Additional file 1. Table S.1 Antibodies used for assessment of cellular composition of CART products and of PB samples of patients after CART treatment. Table S.2 Characterization of the leukapheresis and the HD-CAR-1 product by conventional flow cytometry. Table S.3 HD-CAR-1 adverse events according to CTCAE. Figure S.1 Structure of the RV-SFG.CD19.CD28.4-1BBzeta retroviral vector. Figure S.2 Additional data on the cellular composition of CART products and of corresponding PB samples. [file 13045_2023_1470_MOESM1_ESM.docx]

**Supplementary material**

| **Supplemental Tables** | |
| --- | --- |
| **Table S.1** | Antibodies used for assessment of cellular composition of CART products and of PB samples of patients after CART treatment |
| **Table S.2** | Characterization of the leukapheresis and the HD-CAR-1 product by conventional flow cytometry. |
| **Table S.3** | HD-CAR-1 adverse events according to CTCAE. |
| **Supplemental Figure** | |
| **Figure S.1** | Structure of the RV-SFG.CD19.CD28.4-1BBzeta retroviral vector. |
| **Figure S.2** | Additional data on the cellular composition of CART products and of corresponding PB samples. |

**Supplemental Table S.1: Antibodies used for assessment of cellular composition of CART products and of PB samples of patients after CART treatment.**

| **Antibodies** | **vendor** | **Cat.no** | **Staining number** |
| --- | --- | --- | --- |
| CD19-BUV496 | BD | 612938 | 1 |
| CAR T detection reagent | Miltenyi | 130-129-550 | 2 |
| CD16-BUV395 | BD | 563784 | 3 |
| CD33 BUV563 | BD | 741369 | 3 |
| CD314 BUV615 | BD | 751305 | 3 |
| CD27 BUV661 | BD | 741609 | 3 |
| CD8 BUV737 | BD | 741850 | 3 |
| CD45 BUV805 | BD | 612891 | 3 |
| CD141 BV421 | Biolegend | 344114 | 3 |
| IgD Pacific Blue | Biolegend | 348223 | 3 |
| CD45RO BV570 | Biolegend | 304225 | 3 |
| CD11c BV605 | Biolegend | 301635 | 3 |
| CD279 BV650 | BD | 564104 | 3 |
| CD56 BV711 | Biolegend | 318336 | 3 |
| TCRab BV750 | BD | 747180 | 3 |
| CD45RA BV785 | BD | 564552 | 3 |
| CD11b BB515 | BD | 564518 | 3 |
| CD3 Spark Blue550 | Biolegend | 344851 | 3 |
| CD38 PerCP | Biolegend | 303519 | 3 |
| CD94 BB700 | BD | 566535 | 3 |
| TCRgd PerCP-efluor710 | Thermo Fisher | 46-9959-41 | 3 |
| CD1c PE-Dazzle 594 | Biolegend | 331531 | 3 |
| CD95 PE-Fire640 | Biolegend | 305657 | 3 |
| ITGB7 PE-Cy5 | BD | 551059 | 3 |
| CD25 PE-Fire700 | Biolegend | 356145 | 3 |
| FcεR1α PE-Cy7 | Invitrogen | 25-5899-42 | 3 |
| CD4 PE-Fire810 | Biolegend | 344677 | 3 |
| CD197 APC | BD | 566762 | 3 |
| CD123 Alexa Fluor 647 | Biolegend | 306023 | 3 |
| CD14 Spark-NIR 685 | Biolegend | 399209 | 3 |
| CD127 APC R700 | BD | 565185 | 3 |
| CD34 APC-Cy7 | Biolegend | 343514 | 3 |
| HLA-DR APC-Fire810 | Biolegend | 307673 | 3 |
| **Other reagents** | | | |
| efluor506 (Fixable viability dye) | Invitrogen | 65-0866-14 | 3 |
| Brilliant Stain buffer plus | BD | 566385 | 3 |

After every staining, cells were washed and centrifuged (5 min at 350g), resuspended in a final volume of 400 µl FB and filtered through a 35 µm mesh (Falcon) before acquisition on a Cytek Aurora flow cytometer (Cytek Biosciences) was performed.

**Supplemental Table S.2: Characterization of the leukapheresis and the HD-CAR-1 product by conventional flow cytometry.**

| **UPN** | **total cells** | **viability (%)** |  | **CD3^+^CD19CAR^+^CARTs (%)** | **CD3^+^CD19CAR^+^CARTs** | **CD3^+^ cells** | **CD4^+^ CARTs** | **CD8^+^ CARTs** | **manufacturing [days]** |
| --- | --- | --- | --- | --- | --- | --- | --- | --- | --- |
| 1 | 274 x 10^6^ | 92% |  | 44% | 122 x10^6^ | 99% | 67% | 29% | 10 |
| 2 | 205 x 10^6^ | 95% |  | 46% | 116 x10^6^ | 94% | 61% | 27% | 10 |
| 3 | 272x 10^6^ | 92% |  | 46% | 1101 x10^6^ | 97% | 67% | 28% | 10 |
| 4 | 363 x10^6^ | 96% |  | 53% | 191 x10^6^ | 94% | 2% | 97% | 10 |
| 5 | 152 x10^6^ | 91% |  | 51% | 78 x10^6^ | 95% | 33% | 64% | 10 |
| 6 | 303 x10^6^ | 94% |  | 65% | 197 x10^6^ | 98% | 19% | 67% | 10 |
| 7 | 266 x10^6^ | 91% |  | 59% | 156 x10^6^ | 99% | 34% | 62% | 10 |
| 8 | 329 x10^6^ | 90% |  | 67% | 220 x 10^6^ | 99% | 70% | 27% | 10 |
| 9 | 178 x10^6^ | 88% |  | 45% | 81 x10^6^ | 95% | 29% | 66% | 10 |
| 10 | 344 x10^6^ | 95% |  | 56% | 193 x10^6^ | 96% | 59% | 39% | 10 |
| 11 | 234 x10^6^ | 85% |  | 54% | 127 x10^6^ | 99% | 23% | 73% | 10 |
| 12* | 142 x10^6^ | 98% |  | 39% | 56 x10^6^ | 97% | 44% | 48% | 14 |
| 13 | 1422 x 10^6^ | 98% |  | 55% | 785 x10^6^ | 100% | 36% | 57% | 13 |
| 14 | 220 x10^6^ | 95% |  | 53% | 116 x10^6^ | 94% | 19% | 67% | 10 |
| 15 | 356 x10^6^ | 92% |  | 65% | 231 x10^6^ | 99% | 88% | 10% | 13 |

PBMCs from the leukapheresis product as well as the final HD-CAR-1 product were analyzed by flow cytometry. Flow cytometry was performed on a FACS Canto Flow Cytometry Cell Analyzer (BD Bioscience). Data were analyzed with DIVA software Version 8.0 (BD Bioscience). For immunophenotyping of the PBMCs the following monoclonal antibodies were used: CD3 (clone SK7), CD19 (clone SJ25C1), CD45 (clone 2D1) (all former from BD Bioscience), CD45RA (clone HI100) (eBioscience) and CD197 (CCR7, clone G043H7) (Biolegend). LIVE/DEAD fixable NEAR-IR (Thermo Scientific) was used to detect dead cells. For immunophenotyping of the manufactured HD-CAR-1 product, the following additional monoclonal antibodies were used: CD4 (clone SK3), CD8 (clone SK1), CD14 (clone MOP9), and CD34 (clone 8G12). For detection of CD19-directed CAR specific cells, the biotinylated CD19 CAR Detection Reagent and the Anti-Biotin antibody (both Miltenyi Biotec) were used. Staining was performed according to the manufacturer’s instruction. After staining, the cells were fixed with 4% paraformaldehyde (PFA) (Morphisto) and stored at 4°C until measurement. Compensation was based on PBMCs and non-transduced control (NTC) cells and fluorescence minus one control (FMO) were performed for CD45RA and CD197 as well as for CD19-positive CARTs. CD45 vs. CD3 was used to gate for CD3 T cells from which CD19 CARTs were selected. Cell counting was performed with the automated cell counting system LunaFL (Logos Biosystems). In the final product no B cells or CD34-positive stem cells were detected.

*CARTs production was performed twice. Due to a low T cell number in the leukapheresis product, required dose was not achieved within one production. CARTs from both productions were administered to the patient.

| **Adverse event** | **Grade I** | **Grade II** | **Grade III** | **Grade IV** | **Grade V** |
| --- | --- | --- | --- | --- | --- |
|  | **patients (%)** | **patients (%)** | **patients (%)** | **patients (%)** | **patients (%)** |
| Anxiety | 1 (8) | - | - | - | - |
| Arthralgia | 1 (8) | - | - | - | - |
| Blurred vision | 2 (15) | - | - |  | - |
| Confusion | 1 (8) | - | - | - | - |
| Constipation | 1 (8) | - | - | - | - |
| Cough | 2 | - | - | - | - |
| Delirium | 1 (8) | - | - | - | - |
| Dry eyes | 1 (8) | - | - | - | - |
| Dyspnea | 1 (8) | - | - | - | - |
| Fatigue | 1 (8) | - | - | - | - |
| Generalized muscle weakness | 1 (8) | - | - | - | - |
| Headache | 2 (15) | - | - | - | - |
| Hypophosphatemia | - | 1 (8) | - | - | - |
| Hypotension | 1 (8) | - | - | - | - |
| Upper respiratory infection | - | 2 (15)* | - | - | - |
| Infection | 1 (8)^#^ | - | - | - | - |
| Kidney injury | - | - | - | 1 (8)^§^ | - |
| Heart failure | 1 (8) | - | - | - | - |
| Malaise | 1 (8) | - | - | - | - |
| Multi organ failure |  | - | - | - | 1 (8)^†^ |
| Nausea | 2 (15) | - | - | - | - |
| Neck pain | 1 (8) | - | - | - | - |
| Oral mucositis | 1 (8) | - | - | - | - |
| Pain lower back |  | 1 (8) | - | - | - |
| Palpitations | 1 (8) | - | - | - | - |
| Paresthesia | 2 (15) | - | - | - | - |
| Pleuritic pain | 1 (8) | - | - | - | - |
| Pruritus | 1 (8) | - | - | - | - |
| Bacteremia | - | 1 (8)° | - | - | - |
| Insomnia | 1 (8) | - | - | - | - |
| TSH increased | 1 (8) | - | - | - | - |
| Vertigo | 4 (31) | - | - | - | - |

**Supplemental Table S.3:** **HD-CAR-1 adverse events according to CTCAE.**

* no pathogen identified; ^#^ respiratory syncytial virus (RSV); ^§^ kidney failure requiring dialysis attributed to progressive disease (PD) with concomitant tumor lysis syndrome, not considered dose-limiting toxicity (DLT). °*Staphylococcus epidermidis*; ^†^: death by multi-organ failure doe to a septic shock caused by *E. coli* due to an enterocolitis most likely triggered by an exacerbation of a GvHD of the gut after alloSCT (due to GvHD as pre-existing condition not considered DLT). TSH: thyroid stimulating hormone.

**Supplemental figures**

**Supplemental Figure S.1: Structure of the RV-SFG.CD19.CD28.4-1BBzeta retroviral vector**

The retroviral vector used for CART manufacturing within the HD-CAR-1 trial is based on replication incompetent Moloney murine leukemia (Mo-MuLV) virus. The single chain variable fragment (scFv) targeting CD19 was cloned from the FMC63 antibody. The signal peptide of the human immunoglobulin heavy chain was added to the 5’ end of the CD19 scFv gene. The hinge domain consists of the CH2CH3 domains of the human IgG1 immunoglobulin heavy constant region. The variable regions of immunoglobulin heavy and light chains were generated by reverse transcription of hybridoma RNA with variable region-specific primers. The transmembrane and intracellular domains of CD3ζ, CD28 and 4-1BB (CD137) were derived from Jurkat T cell DNA. The CD19.CAR-CD28/CD137ζ cassettes were cloned into the retroviral vector backbone.

**
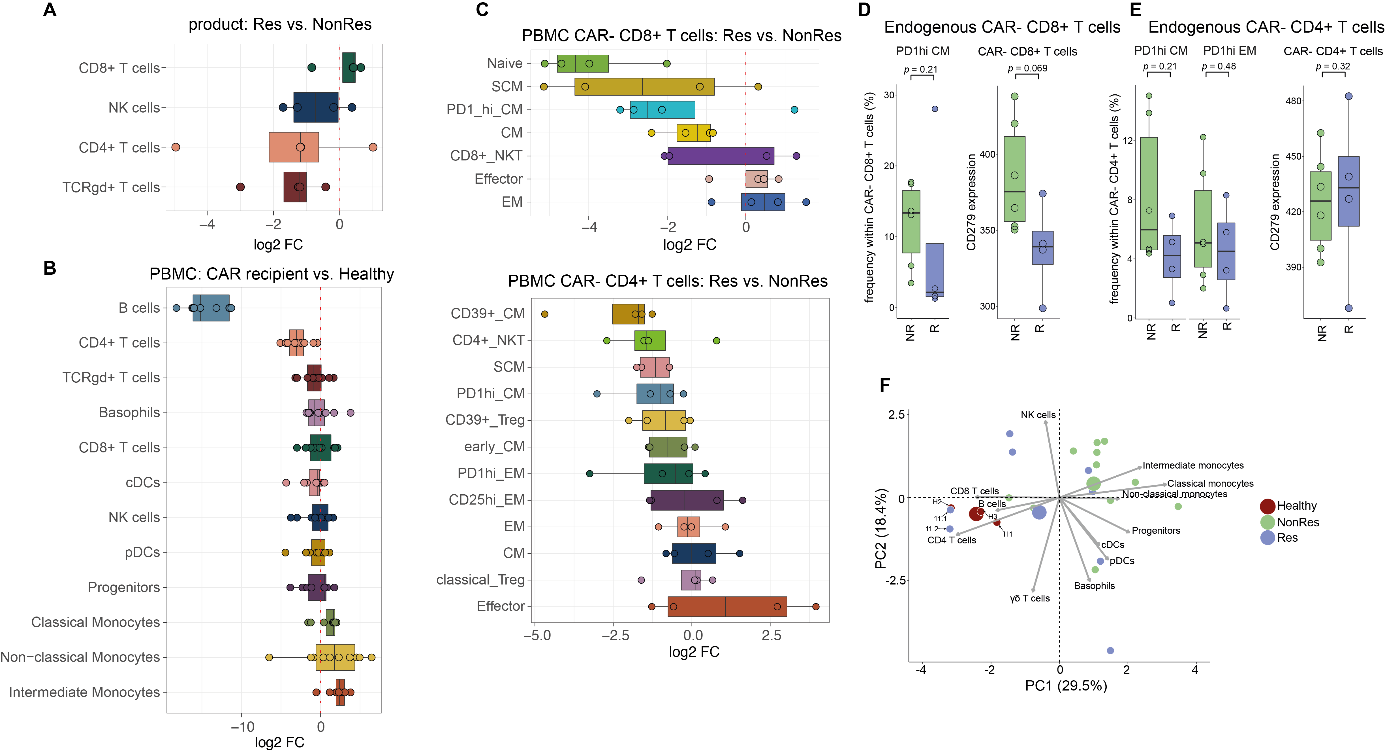
**

**Supplemental Figure S.2: Additional data on the cellular composition of CART products and of corresponding PB samples.**

(A) Boxplots indicate differential abundances of cell populations within the CART product (displayed in main **Figure 5.A**) between responders and non-responders. Positive log_2_ fold changes indicate that a respective population is more abundant in responders, whereas negative log_2_ fold changes indicate that the population is more abundant in non-responders.

(B) Boxplots indicate differential abundances of cell populations within the PBMC samples of CART recipients after treatment and healthy donors (displayed in main **Figure 6.A**). Here, differential abundance between CART recipients and healthy donors was calculated and depicted. Positive log_2_ fold changes indicate that a respective population is more abundant in CAR recipients, whereas negative log_2_ fold changes indicate that the population is more abundant in healthy donors.

(C) Cell populations from main **Figure 6.D** were used and binned into CAR- and CAR+ CD8+ or CD4+ T cells respectively. Boxplots display differential abundance of CAR- CD8+ T cells (top) or CAR- CD4+ T cells (bottom) retrieved after CART treatment between responders and non-responders. Positive log_2_ fold changes indicate that a respective population is more abundant in responders, whereas negative log_2_ fold changes indicate that the population is more abundant in non-responders.

(D) Boxplots indicating the frequency of PD1hi CM cells within CAR- CD8+ in responders and non-responders (left) and the mean intensity of PD1 expression in CAR- CD8+ T cells (right).

(E) Boxplots indicating the frequency of PD1hi CM cells within CAR- CD4+ in responders and non-responders (left) and the mean intensity of PD1 expression in CAR- CD4+ T cells (right).

(F) Principal component analysis (PCA) of all patient samples and three healthy donors. Cell type frequencies were calculated for each sample and used as input for the PCA. Blue circles represent samples from responders. Green circles represent samples from non-responders. Red circles represent samples from healthy donors. The three larger circles indicate the midpoint of the respective group. Gray arrows indicate the variables. Notably, long term remission patient, i.e. UPN #11, clustered with healthy donors.
